# Genetic Diversity of *Mycobacterium tuberculosis* Isolates from Assam, India: Dominance of Beijing Family and Discovery of Two New Clades Related to CAS1_Delhi and EAI Family Based on Spoligotyping and MIRU-VNTR Typing

**DOI:** 10.1371/journal.pone.0145860

**Published:** 2015-12-23

**Authors:** Kangjam Rekha Devi, Rinchenla Bhutia, Shovonlal Bhowmick, Kaustab Mukherjee, Jagadish Mahanta, Kanwar Narain

**Affiliations:** Regional Medical Research Centre, N.E. Region (Indian Council of Medical Research), Post Box #105, Dibrugarh 786 001, Assam, India; St. Petersburg Pasteur Institute, RUSSIAN FEDERATION

## Abstract

Tuberculosis (TB) is one of the major public health concerns in Assam, a remote state located in the northeastern (NE) region of India. The present study was undertaken to explore the circulating genotypes of *Mycobacterium tuberculosis* complex (MTBC) in this region. A total of 189 MTBC strains were collected from smear positive pulmonary tuberculosis cases from different designated microscopy centres (DMC) from various localities of Assam. All MTBC isolates were cultured on Lowenstein-Jensen (LJ) media and subsequently genotyped using spoligotyping and 24-loci mycobacterial interspersed repetitive units-variable number of tandem repeats (MIRU-VNTR) typing. Spoligotyping of MTBC isolates revealed 89 distinct spoligo patterns. The most dominant MTBC strain belonged to Beijing lineage and was represented by 35.45% (n = 67) of total isolates, followed by MTBC strains belonging to Central Asian-Delhi (CAS/Delhi) lineage and East African Indian (EAI5) lineage. In addition, in the present study 43 unknown spoligo patterns were detected. The discriminatory power of spoligotyping was found to be 0.8637 based on Hunter Gaston Discriminatory Index (HGDI). On the other hand, 24-loci MIRU-VNTR typing revealed that out of total 189 MTBC isolates from Assam 185 (97.9%) isolates had unique MIRU-VNTR profiles and 4 isolates grouped into 2 clusters. Phylogenetic analysis of 67 Beijing isolates based on 24-loci MIRU-VNTR typing revealed that Beijing isolates from Assam represent two major groups, each comprising of several subgroups. Neighbour-Joining (NJ) phylogenetic tree analysis based on combined spoligotyping and 24-loci MIRU-VNTR data of 78 Non-Beijing isolates was carried out for strain lineage identification as implemented by MIRU-VNTR*plus* database. The important lineages of MTBC identified were CAS/CAS1_Delhi (41.02%, n = 78) and East-African-Indian (EAI, 33.33%). Interestingly, phylogenetic analysis of orphan (23.28%) MTBC spoligotypes revealed that majority of these orphan isolates from Assam represent two new sub-clades Assam/EAI and Assam/CAS. The prevalence of multidrug resistance (MDR) in Beijing and Non-Beijing strains was found to be 10.44% and 9.01% respectively. In conclusion, the present study has shown the predominance of Beijing isolates in Assam which is a matter of great concern because Beijing strains are considered to be ecologically more fit enabling wider dissemination of *M*. *tuberculosis*. Other interesting finding of the present study is the discovery of two new clades of MTBC isolates circulating in Assam. More elaborate longitudinal studies are required to be undertaken in this region to understand the transmission dynamics of MTBC.

## Introduction

In spite of the implementation of the Revised National Tuberculosis Control Program (RNTCP), the burden of tuberculosis (TB) in India is still the highest which is an important public health concern [1]. India being the second highest populated country in the world, alone accounted for 26% of the world’s tuberculosis cases [2]. According to the World Health Organization (WHO) estimate [2] the largest number of incident TB cases (2.0–2.3 million) are in India.

In order to understand the epidemiology of TB globally as well as locally, the use of molecular typing methods has become imperative not only for understanding genetic diversity and population structure of *Mycobacterium tuberculosis* complex (MTBC) but also for carrying out supervision and monitoring of TB control programs and for understanding TB epidemics [3]. In recent epidemiological studies, most currently used state-of-the-art molecular genotyping techniques are spoligotyping and mycobacterial interspersed repetitive units-variable number of tandem repeats (MIRU-VNTR) typing [4]. Numerous studies from India [5–20] have already reported characterization of MTBC isolates using spoligotyping which is PCR-based reverse hybridization blotting technique based on polymorphism in the presence or absence of “43 spacers” in the Direct Repeat (DR) locus of MTBC genome. Although spoligotyping is quick and convenient genotyping method it has some limitations due to frequent homoplasy and recent studies have pointed out that sometimes phylogenetically unrelated MTBC strains tend to have same spoligotype patterns as a result of convergent evolution or due to independent mutational changes [21]. On the other hand, MIRU-VNTR typing method is more discriminatory and is less prone to homoplasy and has thus been increasingly used along with spoligotyping as a better tool for assessment of tuberculosis progression among infected associates [22]. Therefore, we combined these two typing methods in the present study. After the introduction of an optimized 12-loci and 15-loci MIRU-VNTR typing panels, currently, 24-loci MIRU-VNTR set is being used for best possible discrimination of strongly related strains [23]. A number of studies have been reported from India based on 12-loci MIRU-VNTR typing method [13, 14, 20, 24, 25], however, only a few studies are available from India which have used 24-loci MIRU-VNTR typing method [11, 26]. Till date there is no published report of molecular genotyping study of MTBC based on combined analysis of spoligotyping and MIRU-VNTR typing from the state of Assam located in the northeastern (NE) region of India. In this study 189 MTBC isolates from the state of Assam were studied using spoligotyping along with 24-loci MIRU-VNTR typing to better understand the diversity of MTBC isolates and to find the value of different MIRU-VNTR loci for their ability and usefulness to characterize MTBC isolates from this region.

## Materials and Methods

### Ethical statement

This study was approved by the Ethical Committee of Regional Medical Research Centre, Dibrugarh, (Indian Council of Medical Research, NE Region). Written informed consent was sought from all the participants who donated sputum prior to their inclusion in the study. On behalf of the children, consent was taken from their parents or guardians. Patients whose sputum samples were AFB positive were referred to the nearest DOTs (Directly Observed Treatment-Short course centre) centre for treatment.

### Bacterial isolation, culture and identification

In this study a total of 189 isolates of *M*. *tuberculosis* complex were selected for genotyping. In order to be representative of MTBC strains circulating in Assam we collected AFB positive sputum samples over a period of 3 years (September, 2010 to December, 2013) from 14 different designated microscopy centre (DMC) of Assam. All clinical samples were decontaminated using modified Petroff’s method, and cultured in conventional Lowenstein-Jensen (LJ) media at 37°C for 6 to 8 weeks. MTBC were identified and confirmed by means of standard microbiological and biochemical methods [27].

### DNA extraction

DNA was extracted from fresh sub-cultures of 189 MTBC isolates using cetyl-trimethyl ammonium bromide (CTAB) method [28]. DNA was eluted in TE buffer (pH 8.0) and stored at -80°C until used. DNA purity and concentration was determined spectrophotometrically [29].

### Spoligotyping and MIRU-VNTR typing

Spoligotyping was carried out by amplifying the whole Direct Repeat (DR) region using the commercially available kit (Isogen Biosciences^®^, BV, Maarsen the Netherlands now Ocimum Biosolutions) according to a standardized method described [30] using the designated primers pairs of DRa and DRb (DRa, 5’-GGTTTTGGGTCTGACGAC-3’ (biotinylated 5’ end) and DRb, 5’-CCGAGAGGGGACGGAAAC-3’). GeneAmp^®^ PCR system 9700 of Applied Biosystems was used for DNA amplification. The amplified PCR products were hybridized with nitrocellulose membrane having covalently linked 43 spacer oligonucleotides following the manufacturer’s instructions. The hybridized fragments were identified using enhanced chemiluminescence system (GE Healthcare, UK Ltd., Buckinghamshire, UK). The spoligotypes representation was initially reported as 43 digits binary representation of 43 spacers, one (1) was scored for positive hybridization and zero (0) for negative hybridization.

MIRU-VNTR genotyping was performed by amplifying a standard panel of 24- MIRU-VNTR markers, comprised of 12 mycobacterial interspersed repeat units (MIRU2, MIRU4, MIRU10, MIRU16, MIRU20, MIRU23, MIRU24, MIRU26, MIRU27, MIRU31, MIRU39 and MIRU40,) 3 exact tandem repeats (ETR-A, ETR-B and ETR-C) and MIRU-VNTR loci identified by Queen’s University Belfast group (QUB26, QUB11b and QUB4156) and Mtub group (Mtub04, Mtub21, Mtub29, Mtub30, Mtub34 and Mtub39) [23, 31, 32]. Polymerase chain reaction (PCR) condition was followed as previously described [33]. All PCR products of the MTBC strains were analyzed in Lab Chip (Caliper life sciences Inc., USA) or conventional submerged agarose gel electrophoresis. After sizing of the PCR fragments using appropriate molecular size marker the copy number of various alleles was assigned according to the reference table as given by Supply *et al*. [23]. The PCR experiment was repeated for confirmation in some ambiguous cases. For quality control of spoligotyping and MIRU-VNTR typing experiments DNA samples of *M*. *tuberculosis* H37Rv, *M*. *bovis* BCG, a known Beijing isolate were included as positive controls and sterile distilled water was used as a negative control.

### Drug sensitivity testing (DST)

DST was carried out using conventional proportion method [34] for all first line anti-TB drugs like rifampicin (RIF), isoniazid (INH), streptomycin (STR), ethambutol (EMB), pyrazinamide (PZA) for all 189 MTBC strains.

### Genotype analysis and comparison with databases

SITVITWEB database freely available online (http://www.pasteur-guadeloupe.fr:8081/SITVIT_ONLINE/) [35] was used for identification of spoligotypes based on presence or absence of 43 spacers in the format of June 12, 2015. We also analyzed our data using MIRU-VNTR*plus* web server available at *www*.*miru-vntrplus*.*org* [36, 37] to characterize the MTBC isolates based on spoligotyping and 24-loci MIRU-VNTR typing profile. Spoligotypes were compared with other reference strains present in the above mentioned databases (SITVITWEB and MIRU-VNTR*plus*) for assignment of Shared International Types (SIT) and assigning specific genotypic lineages. Lineage assignment of MTBC isolates was accomplished using Jaccard’s distance matrix and NJ trees based on combined spoligotyping and MIRU-VNTR typing data. For determining the discriminatory power of spoligotyping and MIRU-VNTR typing we used Hunter Gaston Discriminatory Index (HGDI) which was calculated by the formula given below [38]:

$$D=1-\frac{1}{N(N-1)}\sum_{j=1}^{s}x_{j}\left(x_{j}-1\right)$$

where D is the index of discriminatory power, N the number of unrelated strains tested, ‘*s’* the number of different types, and x_j_ the number of strains belonging to the jth type. HGDI was also used to calculate genetic diversity of individual MIRU-VNTR loci.

### Principal Component Analysis

Spoligotyping data from MTBC isolates from Assam along with similar data from Kanpur, Mumbai and other parts of mainland India, Pakistan, Vietnam, Thailand, China, Korea, Japan, Sri Lanka, Uganda, South Brazil, Brazil, Colombia, Turkey, Germany and Ethiopia were subjected to principal component analysis (PCA) to find geographical areas having similar overall spoligopatterns in the reduced multivariate data space without loss of information due to dimensionality reduction. In other words, geographical localities which cluster together will tend to have similar spoligopatterns. Spoligotype data from other regions used in the above analysis were obtained from various sources including the data was also obtained from SITVITWEB database [9, 13, 35, 39–45]. For this analysis a correlation coefficient matrix of the selected geographical areas was generated based on spoligotype frequency data. This correlation matrix was subjected to PCA. In this analysis only few extracted principal components (PCs) explain most of the data variance, thereby eliminating redundant variables and reducing dataset complexity. The first two PCs which explain most of the variation were used to plot the geographical areas in a two dimensional scatter plot. Similarly, PCA was also used to explore clustering pattern of MTBC isolates from Assam and other geographical localities based on the HGDI of individual standard 12-loci MIRU-VNTR alleles. We used a panel of only 12-MIRU-VNTRs for this analysis because of availability of the limited data on 24-MIRU-VNTR alleles from some regions. Varimax rotated first three PCs were used to make a three dimensional scatter plot. Places that will tend to cluster together in the 3D scatter plot will have similarity in the discriminatory power of 12 corresponding MIRU-VNTR alleles. All the statistical analysis was done in SPSS^®^ version 17.

## Results

### Spoligotyping

**Fig 1:**
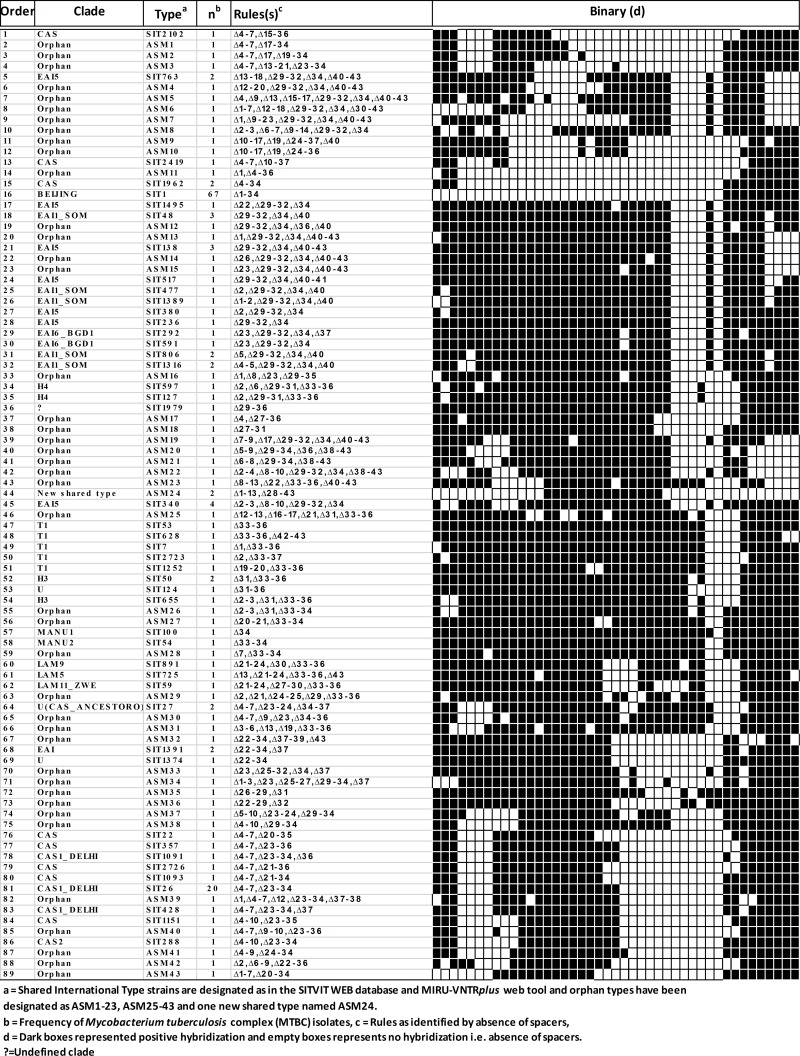
Spoligotype patterns of MTBC circulating in Assam.

Spoligotyping of 189 clinical *M*. *tuberculosis* isolates revealed 89 distinct spoligopatterns (Fig 1). Seventy-six (76) out of 189 clinical isolates were represented by unique patterns whereas remaining 113 (59.78%) isolates were grouped into 13 different clusters. Lineages were identified and assigned using international database MIRU-VNTR*plus* (www.miru-vntrplus.org) and SITVITWEB (http://www.pasteur-guadeloupe.fr:8081/SITVIT_ONLINE/) online tool. In the present study, 44 (23.28%) MTBC isolates had 43 unknown spoligopatterns (i.e. the patterns not yet reported in international database MIRU-VNTR*plus* and SITVITWEB). Out of these 43 unknown spoligo patterns 42 were unique and were therefore designated as orphan strains (ASM 1–23 and ASM 25–43). One unknown spoligotype was found to be represented by two isolates and was therefore considered as new shared type tentatively named ASM24. Table 1 gives the description of spoligotyping defined lineages/sublineages among *M*. *tuberculosis* strains isolated from Assam and their frequencies in study based on SITVITWEB database search. The Beijing lineage (SIT1) representing 35.45% (n = 189) of the total MTBC isolates was found to be the most dominant spoligotype followed by Central Asian-Delhi (CAS1_Delhi) (11.64%). One isolate which was found to belong to SIT1979 could not be assigned to any lineage based on SITVITWEB search. In the present study the discriminatory power of spoligotyping for 189 MTBC isolates from Assam was found to be 0.8637 based on HGDI.

**Table 1:** Distribution of different lineages of MTBC Spoligotypes isolated from Assam in the study based on the classification by SITVITWEB.

Lineage (Family/Sub-family)	No. of isolates	(Prevalence in %, n = 189)
**BEIJING**	**67**	**35.45**
**CAS1_DELHI**	**22**	**11.64**
**EAI5**	**13**	**6.88**
**EAI1_SOM**	**9**	**4.76**
**CAS**	**9**	**4.76**
**T1**	**5**	**2.65**
**H3**	**3**	**1.59**
**EAI6_BGD1**	**2**	**1.06**
**H4**	**2**	**1.06**
**U(CAS_ANCESTORO)**	**2**	**1.06**
**EAI**	**2**	**1.06**
**U**	**2**	**1.06**
**MANU1**	**1**	**0.53**
**MANU2**	**1**	**0.53**
**LAM9**	**1**	**0.53**
**LAM5**	**1**	**0.53**
**LAM11_ZWE**	**1**	**0.53**
**CAS2**	**1**	**0.53**
**Undefined**	**1**	**0.53**
**Orphan**	**44**	**23.28**

**Fig 2:**
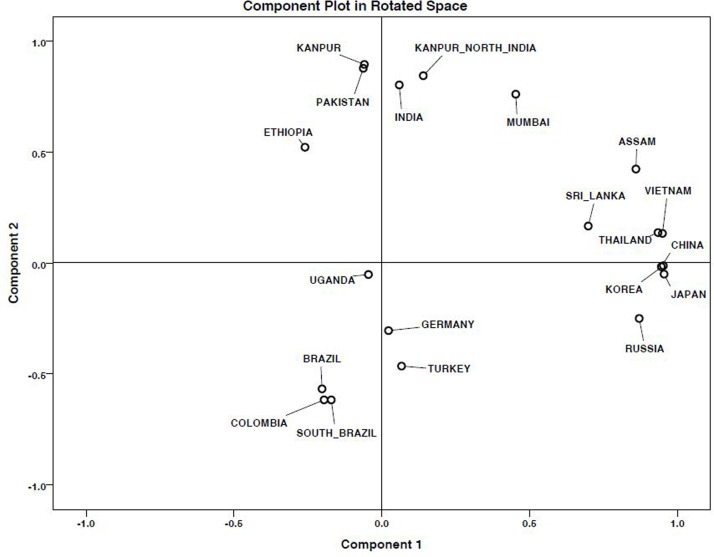
A two dimensional scatter plot based on principal component analysis (PCA). Geographical regions having similarity in patterns of spoligotypes tend to clusters together.

The results of the PCA is given in Fig 2. The 2-D scatter plot based on PC 1 and 2 explained 61.83% of the total variation and revealed that based on overall pattern of spoligotypes, MTBC isolates from Assam were more similar to the MTBC isolates reported from Southeast Asian countries but are quite dissimilar to MTBC isolates from mainland states of India.

### MIRU-VNTR typing of Beijing and Non-Beijing isolates

#### Allelic profiles and discrimination

Table 2 summarizes the allelic profiles and HGDI of the 24-loci MIRU-VNTR in MTBC isolates from Assam. The HGDI of 24-loci MIRU-VNTR revealed that out of 24-loci, twelve loci (MIRU10, MIRU16, MIRU26, MIRU31, MIRU39, Mtub04, Mtub21, Mtub39, QUB11b, QUB26, QUB4156 and ETRA) showed high discriminatory power (HGDI>0.6). Whereas nine loci (MIRU04, MIRU23, MIRU24, MIRU40, Mtub29, Mtub30, Mtub34, ETRB and ETRC) were found to be moderately discriminative (HGDI varies from 0.3 to 0.6), and three loci (MIRU02, MIRU20 and MIRU27) were poorly discriminative (HGDI<0.3).

**Table 2:** The allelic profiles and Hunter Gaston Discriminatory Index (HGDI) of each of the 24 MIRU-VNTR loci in *Mycobacterium tuberculosis* isolates (n = 189) from Assam.

SL.			Copy Numbers of Tandem Repeats: Beijing/Non-Beijing																HGDI		
No.	Alias	Locus	0	1	2	3	4	5	6	7	8	9	10	11	12	13	14	15	Beijing	Non-Beijing	Total
**1**	**MIRU02**	**154**	**0/0**	**0/0**	**65/119**	**0/0**	**2/3**	**0/0**	**0/0**	**0/0**	**0/0**	**0/0**	**0/0**	**0/0**	**0/0**	**0/0**	**0/0**	**0/0**	**0.0588**	**0.0484**	**0.0518**
**2**	**Mtub04**	**424**	**1/1**	**3/5**	**29/74**	**1/14**	**24/17**	**8/4**	**1/5**	**0/1**	**0/0**	**0/0**	**0/1**	**0/0**	**0/0**	**0/0**	**0/0**	**0/0**	**0.6775**	**0.5998**	**0.6461**
**3**	**ETRC**	**577**	**0/0**	**0/0**	**17/75**	**4/6**	**46/41**	**0/0**	**0/0**	**0/0**	**0/0**	**0/0**	**0/0**	**0/0**	**0/0**	**0/0**	**0/0**	**0/0**	**0.4677**	**0.5109**	**0.5513**
**4**	**MIRU04**	**580**	**1/1**	**0/0**	**59/73**	**0/2**	**1/13**	**6/33**	**0/0**	**0/0**	**0/0**	**0/0**	**0/0**	**0/0**	**0/0**	**0/0**	**0/0**	**0/0**	**0.2194**	**0.5617**	**0.4664**
**5**	**MIRU40**	**802**	**0/0**	**5/7**	**10/16**	**43/78**	**6/15**	**3/6**	**0/0**	**0/0**	**0/0**	**0/0**	**0/0**	**0/0**	**0/0**	**0/0**	**0/0**	**0/0**	**0.5586**	**0.5578**	**0.5555**
**6**	**MIRU10**	**960**	**0/0**	**0/0**	**2/6**	**33/14**	**17/27**	**3/33**	**5/15**	**6/12**	**1/10**	**0/5**	**0/0**	**0/0**	**0/0**	**0/0**	**0/0**	**0/0**	**0.6866**	**0.8359**	**0.8259**
**7**	**MIRU16**	**1644**	**0/0**	**0/0**	**5/9**	**7/9**	**19/37**	**29/45**	**7/19**	**0/2**	**0/1**	**0/0**	**0/0**	**0/0**	**0/0**	**0/0**	**0/0**	**0/0**	**0.7155**	**0.7426**	**0.7311**
**8**	**Mtub21**	**1955**	**0/0**	**0/1**	**2/8**	**5/17**	**18/36**	**31/13**	**7/11**	**2/13**	**0/1**	**0/12**	**1/3**	**1/3**	**0/0**	**0/1**	**0/0**	**0/3**	**0.7056**	**0.8537**	**0.8316**
**9**	**MIRU20**	**2059**	**0/0**	**4/6**	**56/107**	**4/5**	**0/1**	**3/3**	**0/0**	**0/0**	**0/0**	**0/0**	**0/0**	**0/0**	**0/0**	**0/0**	**0/0**	**0/0**	**0.2967**	**0.2279**	**0.2514**
**10**	**QUB11b**	**2163**	**0/0**	**1/12**	**13/40**	**4/10**	**5/8**	**16/12**	**24/13**	**0/2**	**2/13**	**1/10**	**0/1**	**0/1**	**0/0**	**1/0**	**0/0**	**0/0**	**0.7779**	**0.8392**	**0.8407**
**11**	**ETRA**	**2165**	**0/0**	**1/4**	**2/6**	**11/21**	**46/47**	**0/0**	**0/0**	**1/8**	**0/4**	**1/5**	**1/4**	**3/22**	**1/1**	**0/0**	**0/0**	**0/0**	**0.5052**	**0.7842**	**0.7084**
**12**	**Mtub29**	**2347**	**0/1**	**0/0**	**5/3**	**0/3**	**14/54**	**48/60**	**0/0**	**0/0**	**0/1**	**0/0**	**0/0**	**0/0**	**0/0**	**0/0**	**0/0**	**0/0**	**0.4441**	**0.5655**	**0.5448**
**13**	**Mtub30**	**2401**	**0/0**	**2/11**	**23/82**	**0/9**	**39/17**	**2/2**	**1/1**	**0/0**	**0/0**	**0/0**	**0/0**	**0/0**	**0/0**	**0/0**	**0/0**	**0/0**	**0.5495**	**0.5192**	**0.5992**
**14**	**ETRB**	**2461**	**0/0**	**2/9**	**53/69**	**5/4**	**2/4**	**5/32**	**0/3**	**0/1**	**0/0**	**0/0**	**0/0**	**0/0**	**0/0**	**0/0**	**0/0**	**0/0**	**0.3668**	**0.6080**	**0.5409**
**15**	**MIRU23**	**2531**	**0/0**	**0/0**	**0/4**	**1/3**	**0/1**	**48/59**	**17/44**	**1/5**	**0/1**	**0/0**	**0/5**	**0/0**	**0/0**	**0/0**	**0/0**	**0/0**	**0.4283**	**0.6361**	**0.5757**
**16**	**MIRU24**	**2687**	**0/0**	**55/74**	**9/16**	**3/31**	**0/0**	**0/1**	**0/0**	**0/0**	**0/0**	**0/0**	**0/0**	**0/0**	**0/0**	**0/0**	**0/0**	**0/0**	**0.3107**	**0.5548**	**0.4868**
**17**	**MIRU26**	**2996**	**0/0**	**2/3**	**9/44**	**0/4**	**2/9**	**3/4**	**27/23**	**21/29**	**2/2**	**1/0**	**0/3**	**0/1**	**0/0**	**0/0**	**0/0**	**0/0**	**0.7273**	**0.7751**	**0.7788**
**18**	**MIRU27**	**3007**	**0/0**	**0/0**	**1/5**	**9/11**	**56/104**	**0/1**	**0/0**	**0/0**	**1/0**	**0/0**	**0/1**	**0/0**	**0/0**	**0/0**	**0/0**	**0/0**	**0.2872**	**0.2655**	**0.2725**
**19**	**Mtub34**	**3171**	**0/0**	**3/3**	**0/3**	**13/22**	**48/89**	**3/3**	**0/0**	**0/1**	**0/0**	**0/0**	**0/0**	**0/0**	**0/0**	**0/0**	**0/1**	**0/0**	**0.4518**	**0.4369**	**0.4403**
**20**	**MIRU31**	**3192**	**0/0**	**0/0**	**1/0**	**11/21**	**5/18**	**9/13**	**41/67**	**0/3**	**0/0**	**0/0**	**0/0**	**0/0**	**0/0**	**0/0**	**0/0**	**0/0**	**0.5834**	**0.6403**	**0.6194**
**21**	**Mtub39**	**3690**	**0/0**	**0/6**	**3/8**	**6/8**	**49/51**	**0/11**	**6/24**	**3/10**	**0/1**	**0/1**	**0/1**	**0/0**	**0/1**	**0/0**	**0/0**	**0/0**	**0.4518**	**0.7667**	**0.6803**
**22**	**QUb26**	**4052**	**0/0**	**2/14**	**2/7**	**4/10**	**4/17**	**1/8**	**3/12**	**10/12**	**11/16**	**15/15**	**15/10**	**0/1**	**0/0**	**0/0**	**0/0**	**0/0**	**0.8521**	**0.9020**	**0.8922**
**23**	**QUB4156**	**4156**	**4/1**	**10/49**	**35/26**	**16/29**	**0/11**	**2/5**	**0/0**	**0/1**	**0/0**	**0/0**	**0/0**	**0/0**	**0/0**	**0/0**	**0/0**	**0/0**	**0.6531**	**0.7328**	**0.7401**
**24**	**MIRU39**	**4348**	**0/0**	**1/3**	**11/36**	**16/27**	**38/52**	**1/3**	**0/0**	**0/0**	**0/1**	**0/0**	**0/0**	**0/0**	**0/0**	**0/0**	**0/0**	**0/0**	**0.6029**	**0.6866**	**0.6622**

The HGDI of individual alleles of 24-loci MIRU-VNTRs in Assam based on stratified analysis of Beijing and Non-Beijing strains revealed that discriminatory power of various MIRU-VNTR alleles was lower in Beijing isolates as compared to Non-Beijing isolates. However, seven alleles such as MIRU02, MIRU20, MIRU27, MIRU40, Mtub04, Mtub30 and Mtub34 showed slightly higher discriminatory power in Beijing MTBC isolates from Assam as compared to Non-Beijing isolates from Assam (Table 2).

Out of total 189 MTBC isolates from Assam 185 isolates were found to have unique MIRU-VNTR profiles i.e. there was absence of any clustering of 185 isolates. Four isolates were grouped into two clusters. The discriminatory power of combined 24-loci MIRU-VNTR panel and combined 15-loci MIRU-VNTR panel was same (HGDI: 0.9999 for both). However, for 12-loci MIRU-VNTR panel the HGDI was 0.9985 (Table 3).

**Table 3:** Hunter Gaston Discriminatory Index (HGDI) and cluster results based on MIRU-VNTR loci analysis of 189 *Mycobacterium tuberculosis* complex (MTBC) isolates from Assam.

Typing Method	Total No. of patterns	No. of uniquetypes	Total no.of clusters	Total no. of isolates in clusters (%)	Maximum no. of isolates in a cluster	HGDI
24 loci MIRU-VNTR	187(N = 189)	185	2	4(2.11)	2	0.9999
15 loci MIRU-VNTR	187(N = 189)	185	2	4(2.11)	2	0.9999
12 loci MIRU-VNTR	172(N = 189)	161	11	28(14.81)	5	0.9985

#### Phylogenetic analysis

**Fig 3:**
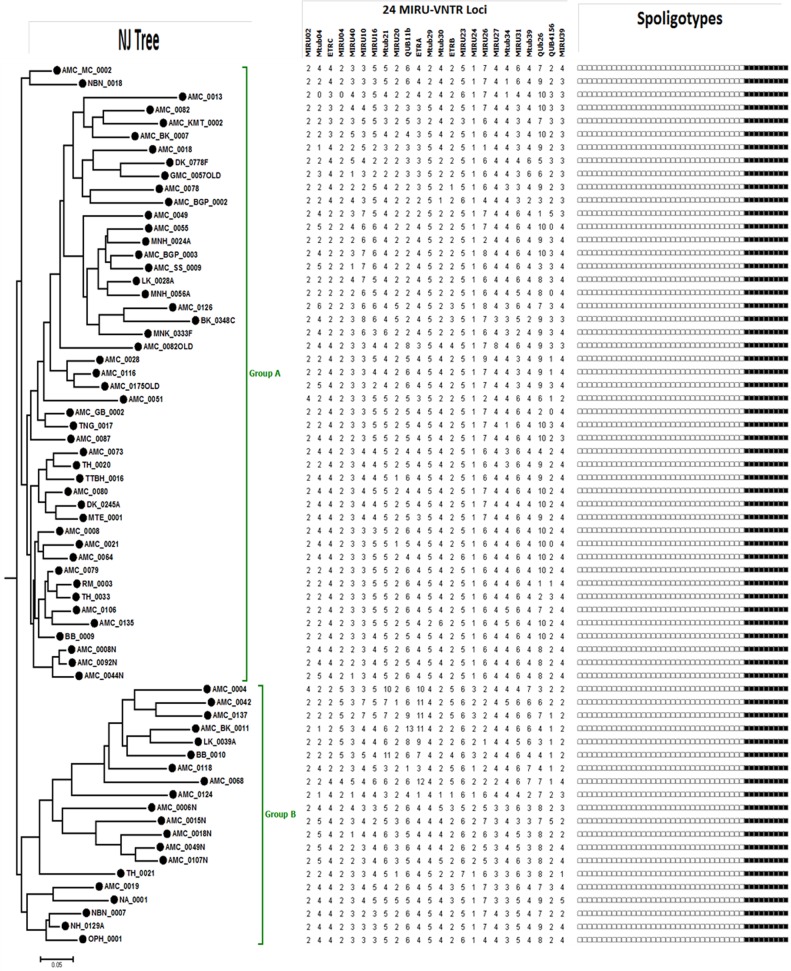
Neighbour-Joining (NJ) tree showing genetic diversity of 67 Beijing isolates from Assam based on 24 MIRU-VNTR loci. Two major clusters (Group A and Group B) and their sub-lineages can be seen from this figure.

In this study we also determined the genetic relatedness among 67 Beijing isolates of MTBC based on Neighbour-joining (NJ) tree constructed using 24-loci MIRU-VNTR data based on Jaccard’s distance matrix (Fig 3). Phylogenetic analysis clearly shows that Beijing isolates from Assam represent two major groups (Group A and Group B) each comprising of several sub-groups.

**Fig 4:**
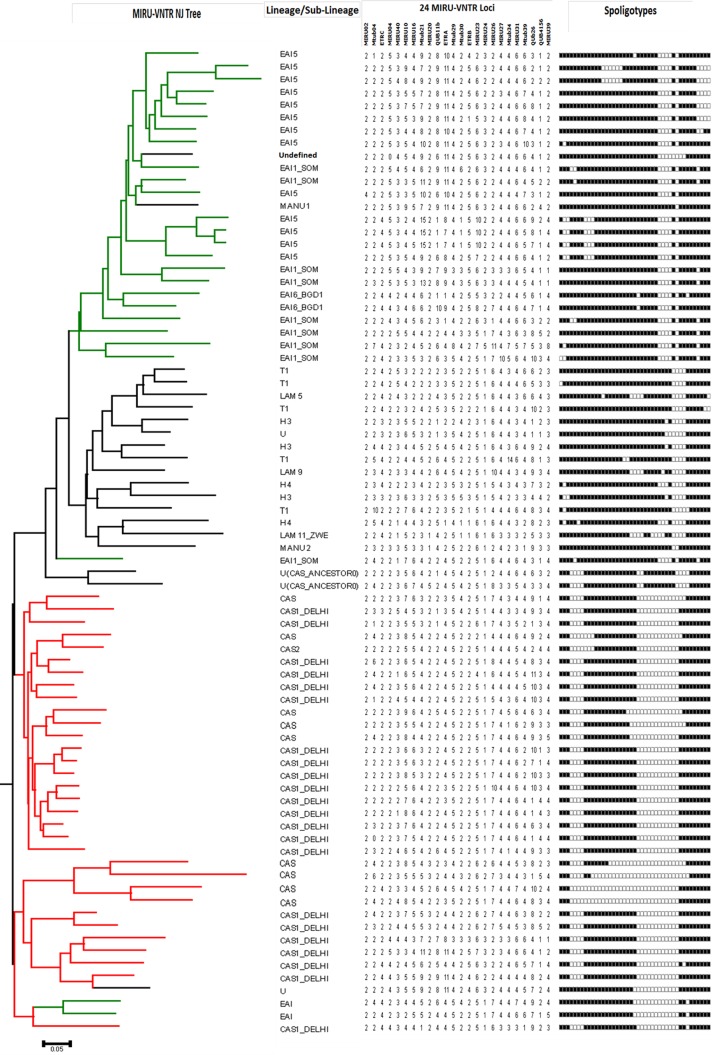
Genotypic diversity of 78 Non-Beijing and Non-orphan MTBC isolates from Assam. A MIRU-VNTR based dendrogram was generated using NJ tree analysis. *M*. *tuberculosis* strain lineages were identified using MIRU-VNTR*plus* identification database. 24-loci MIRU-VNTR alleles and spoligotypes from 78 isolates are also represented along with MIRU-VNTR NJ tree.

We also analyzed 78 Non-Beijing isolates (excluding orphan isolates) obtained in the present study for strain lineage identification based on 24-loci MIRU-VNTR and spoligotyping. MTBC lineage distribution was determined by constructing a NJ tree based on 24-loci MIRU-VNTR and spoligotyping. This analysis was achieved by submitting the MTBC isolate genotypes to the MIRU-VNTR*plus* database which in turn carried lineage prediction of submitted isolates by comparing to MIRU-VNTR and spoligotyping profiles of their reference strains (Fig 4). This analysis revealed that 32 (41.02%) out of 78 isolates belong to Central-Asian (CAS) lineage, out of which 22(68.8%; n = 32) belong to CAS1-Delhi. Another major lineage in this group was East-African-Indian (EAI), 33.33% (n = 78). Among EAI lineage the EAI5 (50%, n = 26) was most prominent. Other minor lineages include: T1 (5), H3 (3), H4 (2), U (2), U(CAS_ANCESTORO) (2), MANU1 (1), MANU2 (1), LAM5 (1), LAM9 (1), LAM11_ZWE (1) and undefined (1).

**Fig 5:**
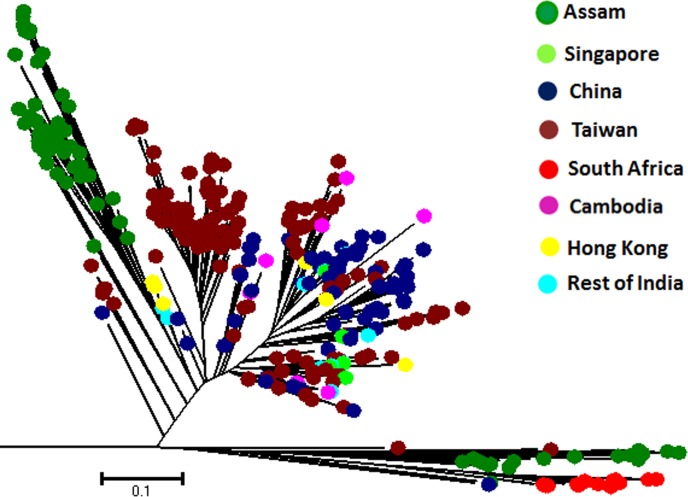
Comparison of Beijing MTBC isolates from Assam with Beijing isolates from other countries (Singapore, Cambodia, Hong Kong, China, Taiwan, South Africa and rest of India) where Beijing isolates are also reported. The radiation tree was constructed based on 24-MIRU-VNTR loci data using Neighbour-Joining method. Beijing isolates from Assam group into two well defined clusters, one major MTBC cluster which is close to some Beijing strains from Hong Kong, China, Taiwan and rest of India whereas the Beijing strains in the minor cluster were phylogenetically more close to a few Beijing strains from South Africa and Taiwan.

In this study we also compared phylogeographic diversity of MTBC isolates belonging to Beijing family from Assam with similar strains from Singapore, Cambodia, Hong Kong, China, Taiwan, South Africa and rest of India where these strains are also reported. The results of this analysis are shown as a NJ radiation tree (Fig 5) which was constructed using 24-MIRU-VNTR alleles. Beijing isolates from Assam grouped into two distinct clusters, one major cluster formed a distinct lineage whereas the Beijing strains in the minor cluster were close to some strains from South Africa and Taiwan.

**Fig 6:**
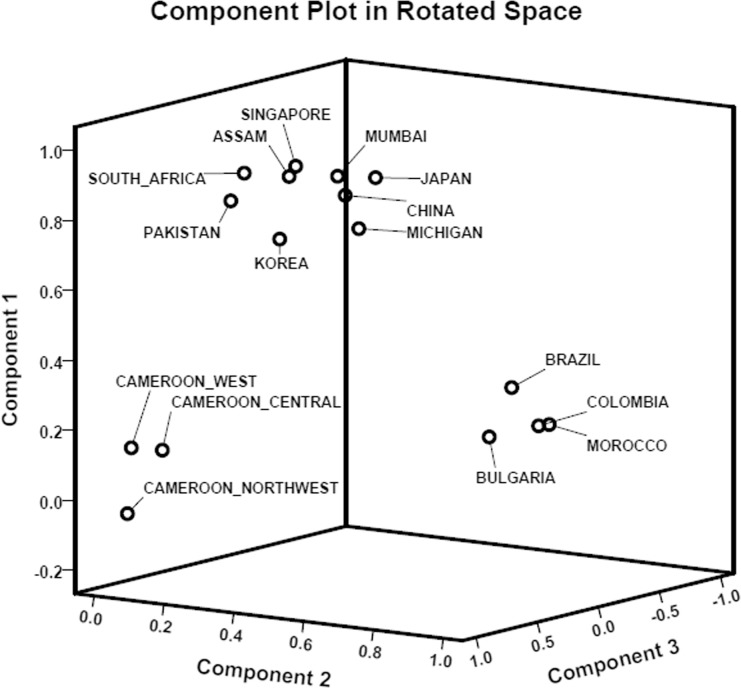
A three dimensional scatter plot based on principal component analysis (PCA) showing relatedness of different geographical regions based on their similarity in patterns of 12-loci MIRU-VNTR diversity in MTBC from Assam and other geographical localities. Different geographical areas were clustered in three groups and the state of Assam clustered with other Asian countries.

In order to explore the similarity in the MTBC strains based on the discriminatory power of individual MIRU-VNTR alleles we carried out PCA analysis. The results are given in the Fig 6. The first three PCs explained about 84.2% of the total variance in the dataset. The 3D scatter plot based on the first three PCs revealed three major clusters of geographical localities. From this figure it is clear that MTBC isolates from Assam cluster with MTBC isolates from other Asian countries. MTBC isolates from Cameroon formed a separate cluster. Similarly, MTBC isolates from South American countries (Brazil and Colombia) and European countries (Bulgaria and Morocco) formed a separate cluster.

### MIRU-VNTR typing of orphan strains

**Fig 7:**
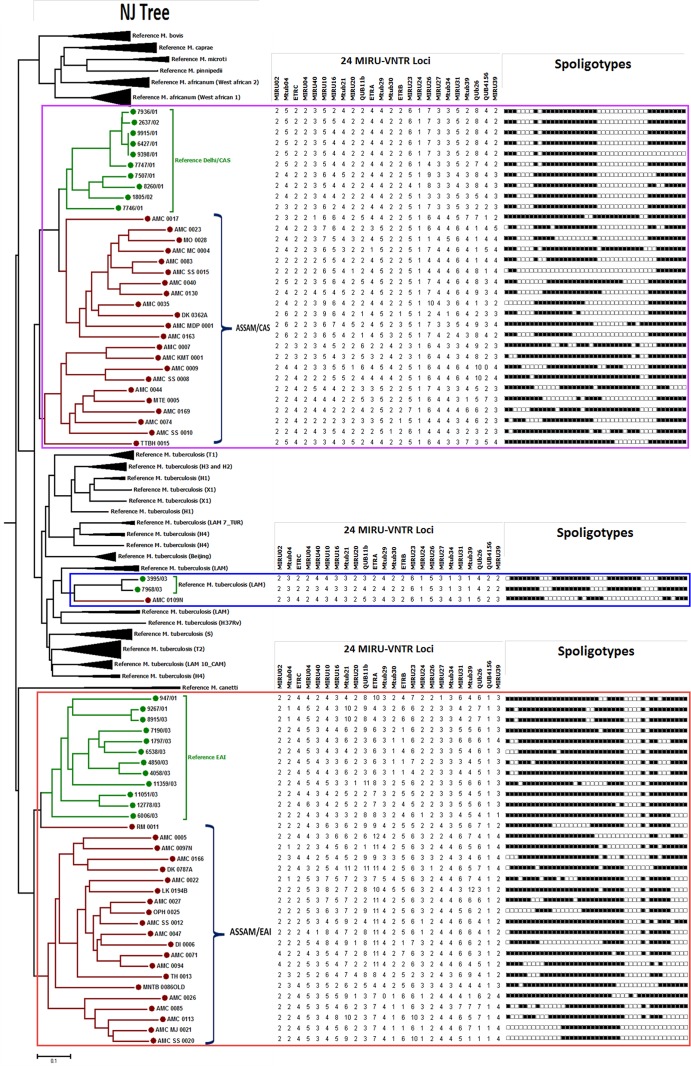
Fig 7 showing phylogenetic relationship of orphan strains of MTBC from Assam with reference MTBC isolates available at the database MIRU-VNTR*plus*. The NJ tree was constructed using 24-loci MIRU-VNTR data. 24-loci MIRU-VNTR alleles and spoligotypes from 44 isolates are also represented along with MIRU-VNTR NJ tree. This phylogenetic tree clearly shows that the orphan strains of MTBC from Assam represent two new well defined sub-clades namely Assam/EAI and Assam/CAS.

In order to understand the genetic affiliation of MTBC orphan spoligotypes discovered in the present study we carried out a phylogenetic analysis including all the 44 MTBC orphan isolates with reference MTBC strains present in the MIRU-VNTR*plus* database [36]. The NJ tree was constructed using combined dataset of 24-loci MIRU-VNTR and spoligotypes as implemented by MIRU-VNTR*plus* tree based identification. The result of the above analysis is given in Fig 7, which clearly shows that majority of these orphan MTBC isolates from Assam represent two new sub-clades namely sub-clade one: Assam/EAI which is related to EAI-Clade and sub-clade two: Assam/CAS which is related to Delhi/CAS clade. One orphan strain clustered with the LAM family of the reference MTBC strain.

### Status of Multidrug-resistance TB (MDR-TB)

The drug susceptibility testing study revealed that 18 (9.52%) isolates were multidrug-resistant (MDR-TB) i.e. these isolates were resistant to at least rifampicin (RIF) and isoniazid (INH) first line anti-tubercular drugs. Eighty-two (43.38%) isolates were found to be resistant to at least one or more anti-TB drugs. Among the 189 MTBC isolates, 63 (33.33%) isolates were resistant to streptomycin (STR). The lowest resistance was observed against pyrazinamide (PZA) with 11(5.82%) of the total isolates. Prevalence of MDR in Beijing and Non-Beijing strains was 10.44% and 9.01%, respectively. A detailed table of the results for each strain: spoligotyping, clade, VNTR typing, origin region, and results of antibiotic susceptibility is given in S1 Table.

## Discussion

The present study is the first study to give an insight into the genetic diversity of MTBC strains from Assam using simultaneously two classical genotyping methodologies viz., spoligotyping and 24-MIRU-VNTR typing. The state Assam is located in the NE region of India and shares international borders with Bhutan and Bangladesh. Assam has sub-tropical monsoon climate and experiences heavy rainfall and high humidity. India being a vast country having great variety in ethnicity and geographical diversity shows differential predominance of MTBC lineages in northern, southern, western and central regions. In northern part of India CAS1_Delhi is predominant strain whereas in Southern and Central India the dominant strain is EAI1 [13, 46–49]. In a recent study from Mumbai, western part of India, a high percentage of Beijing isolates (23%) was reported from a hospital based study which was associated with high prevalence of MDR-TB status [46]. In the present study, we have found 35.45% clinical isolates of MTBC from Assam belonged to Beijing family followed by CAS1_Delhi (11.64%), EAI5 (6.88%), EAI1_SOM (4.76%), CAS (4.76%) and T1 (2.65%) families. Apart from this, very few isolates belonged to H3, EAI6_BGD1, H4, U, U(CAS_ANCESTORO), EAI, LAM5, LAM9, MANU1, MANU2, LAM11_ZWE and CAS2 families. Several recent studies [50–53] have also reported a significant increase in the frequency of Beijing genotypes and this genotype has also been shown to be associated with increased anti-tubercular drugs resistance [51, 53, 54] moreover these strains have been found to have high adaptability to the host intracellular environment in comparison to other lineages of MTBC [55, 56]. A recent study conducted in Dhaka, Bangladesh which is not far away from our study area shown increase in multi drug resistant MTBC to be associated with Beijing strains [57]. In the present study genetic diversity of MTBC strains belonging to the Beijing family isolated from Assam were further characterized by 24-loci MIRU-VNTR profiling and it was found that the Beijing strains from Assam showed two major lineages and five sub-lineages based on NJ phylogenetic tree based analysis. Which indicates highly diverse strains of Beijing family are circulating in Assam and the present finding supports the hypothesis that Beijing isolates are undergoing continuous evolution and active transmission in Assam. The apparent success and predominance of Beijing strains in Assam may be due to wide variety of reasons including human population movements and proximity of Assam to geographical areas where predominance of Beijing strains is high. The present study also revealed that 44 (23.28%) spoligotypes from Assam were found to be orphan which advocates that a significant number of the MTBC isolates prevalent in India are still not well recognized. Interestingly, the NJ based phylogenetic tree constructed revealed that these orphan isolates in fact represent two new clades Assam/EAI and Assam/CAS related to EAI and Delhi/CAS, respectively.

In our study, it was observed through spoligotyping based lineage identification that five MTBC isolates belonged to T1 family, characterized by the deletion of spacers 33–36. This ‘T1’ ancestor is believed to be the prevailing genotype in Delhi region [13]. It was also interesting to find MTBC isolates belonging to CAS and EAI1_SOM family (4.76%), and is the fourth dominant MTBC strain circulating in Assam, NE region of India. In Somalia, this EAI1_SOM family was first reported, and according to the SITVITWEB database (http://www.pasteur-guadeloupe.fr/tb/bd_myco.html) it was also reported from Europe, Asia, Middle East and South Africa [58]. Other clinically important MTBC isolates found in Assam belonged to MANU family but their prevalence was low. The isolate MANU1 (SIT100) is characterized by the absence of only one spacer (spacer 34), whereas, MANU2 (SIT54) strains have two missing spacers (spacer 33 and 34). MANU strains are more frequently reported from mainland India [59] and it is believed that MANU lineage has originated from India [16]. The PCA unambiguously established that the overall spoligotyping patterns of MTBC isolates from Assam are more or less like MTBC isolates reported from Southeast Asian countries rather than the pattern found in mainland states of India. In rest of India Beijing/Beijing-like strains of MTBC are less prevalent, whereas CAS and EAI are more dominant strains of MTBC. On the other hand, in Assam (NE region, India) as in other Southeast Asian countries Beijing isolates of MTBC are more predominant.

High degrees of variation in the allelic diversity of MIRU-VNTRs are reported among MTBC strains in different geographical locations [13, 25, 26, 60]. In the present study MIRU alleles (MIRU10, MIRU26, Mtub21, QUB11b, and QUB26) showed high discriminatory power (HGDI>0.7). On the other hand, lowest discriminatory power was found in alleles MIRU02 and MIRU27 (HGDI<0.3). An earlier study (Mokrousov *et al*., 2004) has shown that MIRU26 and MIRU10 have high HGDI [61]. A recent study from Mumbai has also reported high discriminatory power for the allele MIRU26 and low discriminatory power for alleles MIRU02 and MIRU27. The above study from Mumbai also reported low discriminatory power for MIRU20 (HGDI: 0.04), however, in our samples MIRU20 showed slightly moderate discriminatory power (HGDI: 0.2514). Moderate discriminatory power for MIRU20 has also been reported from Pakistan (HGDI: 0.5080). From the PCA analysis of MIRU-VNTR alleles it was found that MTBC isolates of Assam cluster with MTBC isolates from other Asian countries. This clustering may be due to high genetic diversity of MTBC isolates from Asian countries.

Recent studies have shown that Beijing lineage of MTBC shows genetic heterogeneity [62] and often various branches can be separated into modern (typical) and ancient (atypical) sub-lineages. Ancient sublineages of Beijing strains of MTBC are predominantly reported from Asian countries like northeastern China, Korea and Japan [63]. It can be presumed that most MTBC isolates of Beijing lineages from Assam may belong to ancient Beijing sub-lineage because in NJ tree (S1 Fig) based on 24-loci MIRU-VNTR analysis Beijing isolates from Assam clustered with some Beijing isolates from Korea belonging to ancient sub-lineage. In the present study Beijing strains from Assam were found to form two distinct clusters on the basis of 24-MIRU-VNTR analysis. Recently [21], it has been suggested that some strains which are traditionally referred to as Beijing strains might actually belong to different phylogenetic lineage, which they have referred to as “Pseudo-Beijing” strains. Thus more elaborate studies are required to characterize such strains keeping in view the mutation dynamics within the DR locus [21].

Our DST results revealed that prevalence of MDR was slightly more among Beijing isolates (10.44%) as compared to non-Beijing isolates. However, this difference in prevalence of MDR-TB was not statistically significant.

### Limitations of the study

The small sample size in the present study could present a potential bias regarding representativeness of the strains of MTBC circulating in Assam. In spite of this limitation the present study showed high diversity of MTBC spoligotypes (89 spoligotypes) circulating in Assam. However, future studies with more sample size will throw more light on the diversity of MTBC in Assam.

The second limitation which needs to be pointed out is regarding usefulness of spoligotyping and MIRU-VNTR typing in phylogenetic analysis. Numerous authors have questioned the reliability of spoligotyping for making phylogenetic inference due to homoplasy [64–66]. However, it has been shown that MIRU-VNTR typing is more reliable because of low rate of homoplasy and moreover it has been suggested that only deep phylogenetic inferences may be unreliable and use of more loci of VNTRs will increase reliability of phylogenetic inference [67]. Thus in the present study we have used 24-loci MIRU-VNTR analysis to maximize the phylogenetic reliability of MIRU-VNTR analysis. None the less, the phylogenetic inference drawn by spoligotyping and MIRU-VNTR typing need to be viewed with caution and further studies like whole genome sequencing of MTBC will be required as corroborative data.

In conclusion, the present study has shown the predominance of Beijing isolates in Assam which is a matter of great concern from the public health point of view because it is widely known that Beijing strains of MTBC have high degree of transmission potential in addition to their association with spread of MDR tuberculosis. Other interesting finding of the present study is the discovery of two new clades of MTBC isolates circulating in Assam. More elaborate longitudinal studies are required to be undertaken in this region to understand the transmission dynamics of MTBC. The information on genetic diversity of MTBC isolates obtained from the present study may prove to be useful baseline information for future epidemiological studies on tuberculosis in Assam and will also be helpful for evaluating TB control programs in this region.

## Supporting Information

S1 Fig Comparison of Beijing MTBC isolates from Assam with Beijing isolates from other countries (Japan, Korea, Singapore, Cambodia, Hong Kong, China, Taiwan, South Africa and rest of India) where Beijing isolates are also reported. The radiation tree was constructed based on 24-MIRU-VNTR loci data using Neighbour-Joining method. Beijing isolates from Assam group into two well defined clusters, one major MTBC cluster which is close to some ancient Beijing strains from Korea.
(TIF)

S1 Table Detailed genotyping (Spoligotyping and 24-MIRU-VNTR typing) and drug-susceptibility testing results of 189 *Mycobacterium tuberculosis* strains isolated from Assam, North-eastern state of India. (XLS)
