# Supplementary figures and images for: Genetic Diversity of Mycobacterium tuberculosis Isolates from Assam, India: Dominance of Beijing Family and Discovery of Two New Clades Related to CAS1_Delhi and EAI Family Based on Spoligotyping and MIRU-VNTR Typing
Source: PLoS One. 2015 Dec 23;10(12):e0145860. doi: 10.1371/journal.pone.0145860 (PMC4689458; doi:10.1371/journal.pone.0145860)

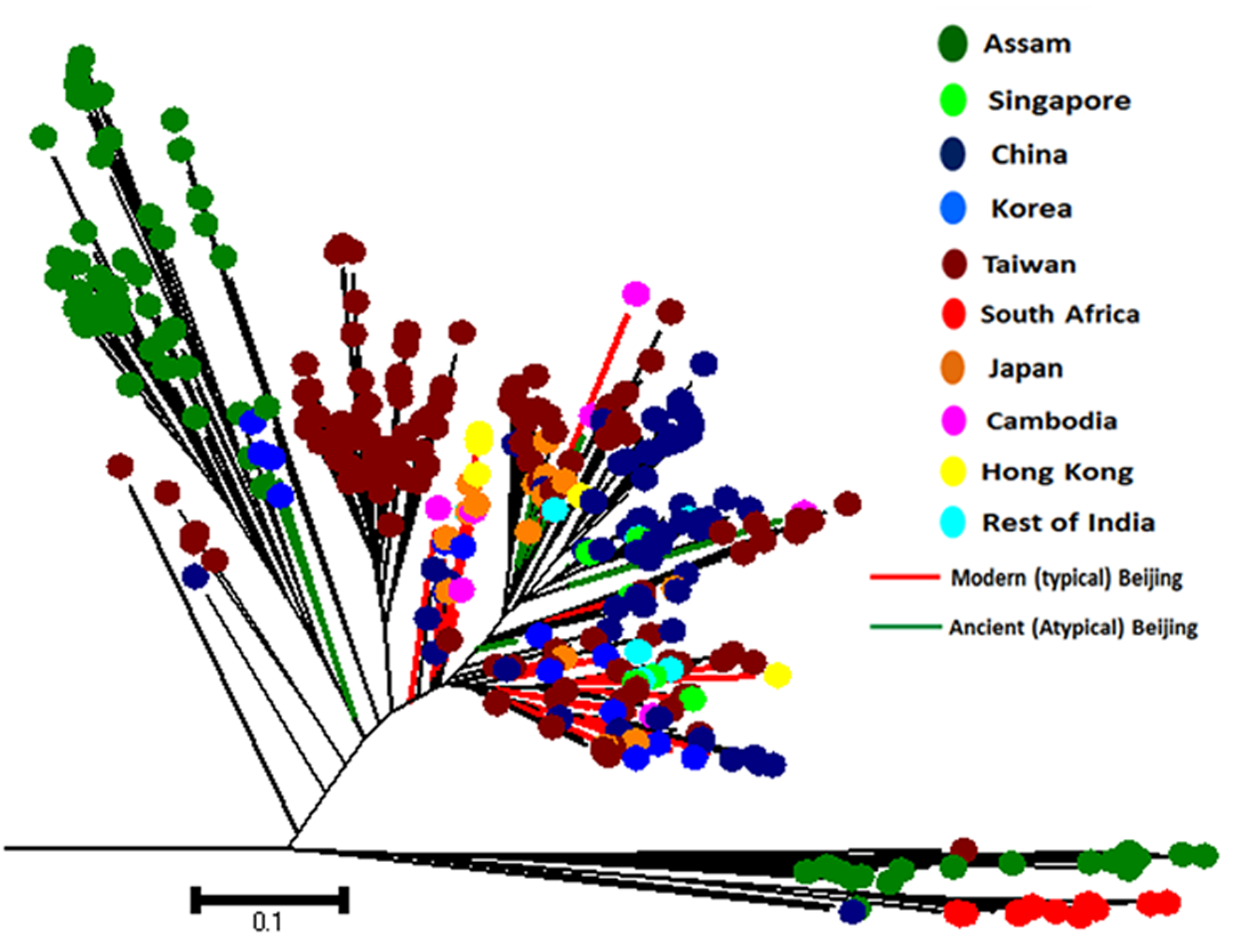

Supplement: S1 Fig — The radiation tree was constructed based on 24-MIRU-VNTR loci data using Neighbour-Joining method. Beijing isolates from Assam group into two well defined clusters, one major MTBC cluster which is close to some ancient Beijing strains from Korea. (TIF) [file pone.0145860.s001.tif]
